# All the right moves: the need for the timely use of hyperbaric oxygen therapy for treating TBI/CTE/PTSD

**DOI:** 10.1186/s13618-015-0028-0

**Published:** 2015-05-28

**Authors:** Kenneth P. Stoller

**Affiliations:** Chief of Hyperbaric Medicine, Hyperbaric Oxygen Clinic of San Francisco, HOCSF/Azzolino CN&IW, 1545 Broadway 1-A, San Francisco, CA 94109 USA

**Keywords:** Football, TBI, Brain Injury, HBOT, Hyperbaric Oxygen, CTE, Encephalopathy

## Abstract

**Background:**

The modern age of hyperbaric medicine began in 1937; however, today few know about hyperbaric oxygen’s effects on the body and medical conditions outside of diving medicine and wound care centers - a serious ethical issue as there are 20 US military veterans committing suicide every day directly related to Traumatic Brain Injury/Post Traumatic Stress Disorder. The problem is not whether hyperbaric oxygen is effective for treating brain injuries, but why the interference in offering this therapy to those who need it.

**Discussion:**

Up against black-boxed anti-depressants that are not efficacious, it should be a “no-brainer” to use a safe, off-label drug, but in the case of military veterans, every suicide might be seen as a tremendous cost saving to certain technocrats. The unspoken rationale is that if the military were to embrace hyperbaric oxygen as the efficacious therapy that it is then current active troops that have suffered injuries will come forward and seek treatment and benefits for their Traumatic Brain Injuries now that they know there is a viable therapy and in so doing troop strength will be decimated. So, to attempt to delay the acceptance of hyperbaric oxygen the Department of Defense has funded faux-studies claiming low pressure room air to be a placebo or sham, and then proclaiming there is no statistical difference between treatment arms and sham or placebo treatment arms. With few who understand hyperbaric medicine there is almost no one to call them on this subterfuge and prevarication. Many peer-reviewed articles have been published in the last decade that demonstrate hyperbaric oxygen is effective in repairing an injured brain even long after that injury took place. One of the most notable showed that blast-induced brain injured war veterans experienced a 15 point IQ increase (p < 0.001).

**Summary:**

Hyperbaric oxygen is an efficacious, benign and humanitarian way to affect brain repair but it has not been adopted because it lacks patent protection and has no large corporate sponsors. It has also met interference because other agendas are present be they the protection of the status quo, myopic budgetary constraints, or perceived liability issues.

## Background

Hyperbaric oxygen therapy (HBOT) saturates the body’s tissues with oxygen using a pressure vessel. HBOT is most often recognized as the treatment for decompression sickness (DCS) or “the bends.” DCS causes significant neurological injury and post-initial injury similar pathophysiology is virtually identical to that caused by trauma. Thus oxygen under pressure has been used to treat neurological injuries since 1937, for 75 years. No one has found a replacement for or substitute treatment for the bends that works as well as oxygen. HBOT results in a 95 % acute treatment cure rate for DCS in all of the navies of the world. Three currently accepted HBOT indications are for neurological conditions, and three are for various kinds of non-healing wounds. Thus there is more evidence for using HBOT for neurological acute and chronic wound treatment, with better clinical outcomes than any other single or combined treatment. The good news that combining HBOT with other therapies that help brain injured patients enhances the effect of those treatments and makes these other therapies less costly, with creating additional recovery in a given patient. Because a patient’s future income is directly tied to recovery after injury, it is important for policy makers to set maximizing patient recovery as a goal to maximize productivity and tax revenue from each given individual.

An increase of one-half to a 1 ½ atmosphere increase will raise the oxygen levels in plasma 7 × -12× normal (700 % to 1200 %) Under this increased pressure oxygen acts like a drug and DNA signaling agent. This treatment’s mechanisms of action simply follow the general gas laws for saturating liquids with a gas, similar to the way Coca-Cola® makes their product. No one yet has found a substitute for oxygen in human physiological processes, and any injury caused by a lack of oxygen can be expected to benefit, with the right oxygen dosage. Saturating with oxygen is a safe procedure, when all of the correct protocols are followed, and significant side effects are extremely rare.

The history of hyperbaric medicine reaches back to the year 1620 when Drebbel developed a one-atmosphere diving bell, and 40 years later Boyle joined forces with Gay-Lussac to develop the General Gas Law. Moving the sands of time to the near present day, the modern age of hyperbaric medicine began in 1937 when Behnke and Shaw used a hyperbaric chamber to treat DCS. However, it was not until 1955 that there was major interest in using hyperbaric oxygenation (HBO) outside of treating DCS. That year, Churchill-Davidson began to use oxygen therapy in a hyperbaric chamber to treat the damage induced by radiotherapy in cancer patients. In 1956, Boerema (Holland) performed the first reported heart surgery on “blue babies” in a hyperbaric chamber. He became the “Father of Hyperbaric Medicine” when he treated a woman who had been badly beaten, was unconscious, and was about to lose her leg. This became the first recorded prevention of an amputation with HBOT, and the woman did well. The next year his famous “Life without Blood” study was published. He referred to the treatment as “oxygen drenching” [1].

In 1962, Sharp & Smith (Scotland) were first to treat carbon monoxide poisoning by HBOT; 1963, Hitchcock testifies before the House Labor Health, Education, and Welfare committee on the need for hyperbaric chambers in surgery and congress appropriates money for building a score of them [2]; 1965, Perrins (United Kingdom) showed the effectiveness of HBOT in osteomyelitis; 1965, Japanese researchers treat the first burn patients; 1966, Saltzman *et al.* (USA) showed the effectiveness of HBOT in stroke patients; 1970, Boschetty and Cernoch (Czechoslovakia) used HBOT for multiple sclerosis (MS); 1971 Lamm (France) used HBOT for treatment of sudden deafness; 1973, Thurston showed that HBOT reduces mortality in myocardial infarction; 1976, Hollbach & Wasserman determine 1.5 ATA (atmospheres absolute) maximizes oxygen content and glucose metabolism in the brain; 1983, first double-blind RCT using HBOT to treat MS; 1987 Jain (Swiss) treats paralysis of stroke with HBOT; 1989, the U.S. Navy discovers that bubbles are gone within 5 min, so while DCS is caused by bubbles the secondary injury cascade is the same as in all brain insults; 1992, Harch treats the first delayed decompression sickness, which lead to the treating “dementia pugilistica” in boxers, cerebral palsy children, and autistic children and nearly 50 neurological conditions in 700 patients. In 1992, Rockswold (USA) conducts first double-blind RCT showing HBOT reduces mortality in acute traumatic brain injury (TBI) by 59 %, the largest single reduction in mortality since the invention of the ambulance. In 2002, US Army study confirms Harch’s HBOT repairs white matter damage in children with cerebral palsy (CP) and a Canadian group shows hyperbaric air (the original treatment for DCS and Mountain Sickness) and HBOT 1.75 effective in treating CP in double-blind RT; 2005, Stoller (USA) first child with fetal alcohol syndrome treated [3] and Thom (USA) finds HBO causes stem cell mobilization; 2007, Harch *et al.* (USA) chronic TBI treated in animal model and in 2009 in military veteran [4]; 2010, Godman discovers HBOT activates 8101 genes, reducing inflammation and increasing growth and repair hormones. In 2011, Stoller treats first retired National Football League (NFL) player treated for CTE [5]; 2012, Harch *et al.* demonstrates blast-induced post-concussion syndrome and post-traumatic stress disorder treatable with HBOT in phase 1 clinical trial [6].

The above is not meant to be a comprehensive timeline nor is this a meta-analysis of HBOT for various conditions, rather an opportunity to understand why a benign yet beneficial therapy has been ignored and even treated with great disdain. Bureaucratic concerns have repeatedly trumped medical and scientific evidence. For someone with training in decision theory and bureaucratic behavior, the problems are very clear. Each time in history when a decision was made about the deployment of hyperbaric oxygen therapy (starting with Behnke’s discovery of oxygen improving the outcomes for DCS), bureaucratic concerns over budget constraints trumped science; to the determent of the persons those bureaucracies were intended to serve. Sometimes it was concerns about costs. Other times it was incorrect assumptions about the impact of the new science on the system.

There is no evidence of pharmaceutical company interference; although, such interference could be inferred after a 1983 study was published in the New England Journal of Medicine showing HBOT to benefit patients with multiple sclerosis. B.H. Fischer, M.D., a tenured professor at New York University, became the principal investigator of a study funded by the National Multiple Sclerosis (MS) Society (which is directly funded by pharmaceutical interests). Apparently, this society had great difficulty accepting the results of the work Dr. Fischer had completed, and multiple revisions were made to weaken the conclusions sufficiently to satisfy the editors of the New England Journal of Medicine. In this double-blind controlled study of patients with advanced chronic disabilities, Fischer found significant improvement in objective measurements, and the treatment effect persisted for at least 1 year [7].

For reasons hard to explain (to a logical mind), this study was never followed up, despite the positive results, and the treatment languished for lack of financial support and sponsorship. Indeed, Fischer lost his position, and his chamber was destroyed.

It is almost always a bureaucratic decision that makes no sense to those who have to enforce it, because if you don’t know the decision logic, one often comes to the conclusion, “it must be fraud.” The reports about HBOT’s impact on patients have never changed. What has changed is we now understand the mechanisms of action and how vital oxygen is to healthy functioning human metabolism.

For example, a 2002 Canadian study [8] found that even room air under relatively low pressure (1.3 atmospheres) improved the clinical outcomes of the children in this thirteen-year-old double-blind randomized study. Ten times more progress was made in Gross Motor Function (GMF) during the 2 months of hyperbaric therapy (while all other therapies were ceased) than during the 3 months of follow-up with OT/PT restarted. The editorial in the Lancet, where the article was published, pointed out that “both groups of children improved substantially with respect to GMF, speech, attention, memory and functional skills.” The Canadian government, which financed the study after being pressured by parents of children with cerebral palsy, falsely claimed the pressurized room air was a placebo and therefore there was no difference between the placebo group and the group of children receiving 100 % oxygen. While this is sadly amusing, it kept HBOT from becoming standard of care for children with CP in Canada and in the United States. This is a gross tragedy and disservice, and not using hyperbaric oxygen therapy to treat these children when their brains are plastic and recovery can be dramatic, leaves them as adults with continued high care costs and lost productivity.

HBOT has truly been the Cinderella of conventional medicine, which means it has been an attractive therapy that has been shown to be efficacious in treating many conditions and yet is treated with derision or ignored at best. Because no patent is possible on oxygen (or any other element), there is no profit to spark a large pharmaceutical interest to prove or promote it. Few know about HBOT’s effects on the body and medical conditions outside of diving medicine and wound care centers. But this has become much more than an issue of lack of marketing and poor public relations. This is now a serious ethical issue as there are now 20 US military veterans committing suicide every day directly related to TBI/PTSD. Suicide losses now exceed combat casualties. Even National Football League (NFL) veterans are starting to commit suicide. The clinical trial called the National Brain Injury Rescue and Rehabilitation project^1^ showed HBOT can virtually eliminate suicidality in this population once they are treated with HBOT, while reducing depression by 51 %. That is a larger and broader effect on depression than anything advertized on television. Yet even on the basis of compassionate use it is not possible to get HBOT paid for to treat TBI even though HBOT has more “on-label” indications for brain injury than any other drug or therapy in medicine. The issue at hand is not whether HBOT is effective for treating TBI/PTSD – HBOT is an effective treatment, the real issue is why the interference, for the time is upon us to expose the obfuscation of this humanitarian therapy - literally people are dying because they are not getting into hyperbaric chambers to breathe oxygen.

## Discussion

In 2009, and again in 2010, Paul Harch, MD, Director of the LSU Hyperbaric Medicine Department, delivered testimony to both the House and Senate Armed Services Committee reminding them that the epidemic of suicides amongst military veterans was most likely due to cocktail of “off—label” antidepressants they were being prescribed – Black-Boxed antidepressants. None of which are approved for treating TBI. Others have delivered this warning as well. The exact FDA warning states: **“Antidepressants increased the risk compared to placebo of suicidal thinking and behavior (suicidality) in children, adolescents, and young adults in short-term studies of major depressive disorder (MDD) and other psychiatric disorders. Anyone considering the use of (***insert name of antidepressant*) **or any other antidepressant in a child, adolescent, or young adult must balance this risk with the clinical need.”**

While antidepressants are modestly effective in reducing the symptoms of severe depression, they increase the brain’s susceptibility to future episodes after they have been discontinued. This fact contradicts pharmaceutical company sponsored research as antidepressants cause neuronal damage and mature neurons to revert to an immature state, both of which may explain why antidepressants also cause neurons to undergo apoptosis (programmed death) [9]. If antidepressants cause death on a micro level then it is much easier to understand why certain patients commit suicide given the human body (macro level) is made up of these cells. So, not only are black-boxed off-label Selective Serotonin Reuptake Inhibitors (SSRI’s) prescribed to a vulnerable population but they are done so without any regard to an ability to metabolize this class of drugs [10] which further exacerbates suicidal behaviors. And then it seems SSRIs actually deplete both catecholamine and serotonin [11], which is exactly what isn’t in a depressed individual’s interest.

Up against black-boxed anti-depressants that are not efficacious, it should be a “no-brainer” to use a safe, off-label drug, i.e., oxygen at hyperbaric doses, to treat those who have received a TBI now with two decades of use treating various neurological conditions (the double-blind RCT by Rockswold [12], showing HBOT effective in treating acute severe TBI, was published in 1992 - decreasing mortality in the acute treatment on severe TBI by 59 %, the largest reduction in mortality since the invention of the ambulance, the use of helicopters in Vietnam for battle casualties, and penicillin for infection). So, what is the problem? What does it take to become “standard-of-care?”

As already pointed out, HBOT is non-patentable. Research on non-patentable or off-patent drugs or with insufficient marketing prospects (orphan drugs) is funded by nonprofit or charitable organizations only. Drugs for which a patent cannot be granted are not being developed, and/or marketed even when they respond to a public health need. Patients, pharmacists, physicians and other caregivers consequently cannot take advantage of potentially effective treatments – they can’t even find out about them.

But while HBOT won’t make any entity large profits, doesn’t it have other monetary incentives? For each active duty brain injured solider returned to duty the lifetime savings to the government is $2.6 million dollars and $2 million for each injured service member returned to work or school. Between 60 and 80 percent of the veterans participating in the National Brain Injury Rescue and Rehabilitation (NBIRR) project are returning to work, duty or school after receiving HBOT. One would think that would move the powers-that-be to action, but it has not, and the reason for that may be because all they can see is what an injured veteran will cost should they stay alive. That is a serious accusation, so to better understand this controversial area on how the Department of Defense (DoD) handles TBI, the NFL provides an example of what takes place on a smaller scale.

For many decades, evidence has linked repetitive traumatic brain injury to long-term neurological problems in many sports. The NFL as the organizer, marketer, and face of the most popular sport in the United States, in which head trauma is a regular occurrence, was aware of the evidence and the risks associated with repetitive traumatic brain injuries and concussions for decades, but apparently ignored and worse actively concealed the information from those who participated in organized football at all levels. That is now the basis of hundreds of lawsuits.

So, what seems to have taken place is the NFL inserted itself into the scientific research and discussion concerning the relationship between concussions and short-term and long-term impairment of the brain. After doing so, the NFL then intentionally and fraudulently mislead present and former players, and all people who reasonably rely upon the NFL’s expertise about its own sport, regarding the short-term and long-term risks posed by concussions and head trauma.

Rather than warn players that they risked permanent brain injury if they returned to play too soon after sustaining a concussion, the NFL actively deceived players, by misrepresenting to them that concussions did not present serious, life-altering risks.

The NFL created the Mild Traumatic Brain Injury Committee (the “MTBI Committee”) in 1994 to research and ameliorates the impact of concussions on NFL players. Notwithstanding the purported purpose of the MTBI Committee, and despite clear medical evidence that on-field concussions led directly to brain injuries with tragic results for players at every level of the sport, the NFL failed to inform its current and former players of the true risks associated with such head trauma and purposefully misrepresented and/or concealed medical evidence on that issue. The NFL also stonewalled on an intervention and therapy that could be helping injured players regardless of whether those injuries were acute or chronic. The author has firsthand experience dealing with the NFL’s 88 Plan in an attempt to get veteran NFL players with dementia HBOT.

The 88 Plan is designed to assist players who are vested under the Bert Bell/Pete Rozelle NFL Player Retirement Plan and who are determined to have dementia. But if a plan member tries to get HBOT using the plan because they have been diagnosed with CTE, they will be told CTE does not cause dementia and therefore HBOT, which treats CTE, will not be a covered benefit. Obviously, that is irrational, but there is often madness behind the reason for not allowing an effective treatment to be utilized by those that need it. In the case of military veterans, for 20 suicides every day might be seen as a tremendous cost saving to certain decision makers. Yet it goes beyond money for if the military embraced HBOT as a viable therapy of TBI/PTSD then many, many troops who are on active duty with TBI/PTSD will come forward once they know there is an efficacious intervention that is recognized by the military. In so coming forward, troop strength could be decimated. This is the fear, and this is what has driven faux research funded by the Department of Defense.

All civilian studies published have been reported as positive even a randomized Israeli study, while all the Department of Defense studies have been consistently negative. One study had to go so far as to say that it was “biologically implausible” for compressed room air to have a healing effect on the brain. That would be called conformational bias if only this were about bias, but this was a deliberate and orchestrated attempt to scuttle HBOT as a recognized therapy for the above reason. Yes, it is illegal to waste tax payer dollars knowing you are creating pseudo-science, but that is beyond the scope to this editorial.

Now, this is truly misanthropic, but no more so than what tobacco corporations do and we still tolerate their malfeasance. In the case of the NFL, the reason for prevaricating about TBI and potential treatments is just a business decision. The exposure of the “hit squad” of the New Orleans’s Saints, where there was a bounty put on players from opposing teams, is a clear example of what kind of business this is about.

A great deal of time has been lost by those who believe hyperbaric oxygen is either a placebo, sham or should be subjected to placebo-controlled studies – or want others to believe this. But oxygen can never be a placebo. HBOT is an FDA-approved drug that affects non-specific biological repair; in fact it is the only non-hormonal FDA-approved treatment known to repair and regenerate human tissue. It does so at a DNA level by activating growth factors and reviving mitochondrial function [13]. The beneficial effects of HBOT apply no matter where a wound or injury is located in the body.

Many peer-reviewed articles have been published in the last decade that demonstrates HBOT is effective at repairing an injured brain even long after that injury took place. One of the most notable was the article published by Harch *et al.* [6] using only one half of the NBIRR protocol (Forty 60 min treatments at 1.5 atmospheres). The blast-induced TBI war veterans experienced a 15 point IQ increase (p < 0.001), 39 % reduction in post concussion symptoms, 30 % reduction in PTSD symptoms and a 51 % decrease in depression. This is all consistent with past-published reports of HBOT in chronic brain injury, including research by the US Army on brain-injured children [14].

The first battle casualty to be treated with HBOT (1.5), and one of the few to be treated was General Patt Maney (retired) for his blast-induced brain injury in Afghanistan. His treatment was ordered after 9 months of therapy at Walter Reed had shown minimal improvement. As a result of his injuries he was non-functional and unable to return to his job, let alone redeploy back to Afghanistan. After HBOT treatment he was discharged from Walter Reed and returned to his civilian job as a Florida state judge.

He received treatment from George Washington University Medical Center at the Tricare Reimbursement rate of $250 per treatment. Counting lost time and hospital costs, his months at Walter Reed making no progress; the DoD spent $400,950, with a permanent disability loss to the service of $1.3 million. Had he received HBOT (at 1.5 atmospheres) earlier, he would have been able to remain on active duty, a savings of $1.3 million, but more importantly, the 5 months of recovery once he began receiving HBOT 1.5 cost $133,650, a savings to the government of $287,300. No other patients were treated at the Walter Reed’s brain injury center, despite the General’s remarkable recovery that everyone on the staff witnessed. The $20,000 for his hyperbaric medical treatment was $12,000 less than what a RAND report states the annual ongoing costs per year of the current treatments for mild-TBI is,^2^ and that is a lot of SSRI’s.

Since every working person represents $1 million in tax revenue over their working life to the government, that government should be interested in, and foster payment for, biological repair of brain injury. Thus every brain injured veteran, all 700,000+ of them, at a cost of $60,000 per year to the economy every year in increased costs and lost productivity, is a $42 billion drain on the economy. Treated, they immediately set about doing what young people do, they begin to form families and create the next American generation. Injured, they are unable to do so, and it is highly likely that these untreated brain injuries are a major cause of our nation’s economic challenges. But bureaucracies do not think logically – their ability to think laterally is limited. When Medicare approved HBOT to treat diabetic foot ulcers at the end of 2002, they only made it available for Wagner III and IV lesions (osteomyelitis and gangrene) so afraid they were of budgetary constraints.

HBOT prevents 75 % of major limb amputations in Wagner III and IV ulcers, but if they had included Wagner II lesions HBOT would be preventing 88 % of amputations. So, the result is there are a lot of unnecessary amputations not because of bad science, but because technocrats were afraid of having a short term budget problem.

## Summary

HBOT is an efficacious, benign and humanitarian way to affect brain repair but it has not been adopted because it lacks patent protection and has no large corporate sponsors. It has also met interference because other agendas are present be they the protection of the status quo, myopic budgetary constraints, or perceived liability issues – after all, when you treat TBI directly the way that HBOT does, the problems creating those TBI’s in the first place are harder to ignore and the unconscious way those problems have been dealt with are harder to deny. It brings the true cost and repercussions of war to the fore, and football itself, after all, is just a form of organized war. This perspective, should it be adopted by the general public, will be a catalyst for change. So, whether that means changing the way football is played to not allowing our “leaders” to guide us into unending military forays for the sack of war-profiting run amuck – all of these sub-terrain issues come into play when a very straight forward and effective therapy tries to assert itself, hence the resistance both on a conscious and subconscious level.

Veterans are considered a threat by the security apparatus in the USA. Is someone thinking that it is better to let 20 potential threats kill themselves every day? This is a dark rabbit hole to go down but the human cost of war is very much a deep rabbit hole and one that many want to keep hidden. Someone is making money when someone else bleeds to death from a cluster bomb. It is that black & white – someone is making money giving dangerous SSRIs for treating TBI. Technocrats rotating between corporation and state are the last people you want making medical decisions, but that is exactly who has been making medical decisions. These are all things to be looked at when asking the question why a therapy like HBOT is being suppressed. If the man on the street understood why certain medical therapies are not available while other dangerous and non-efficacious therapies are favored then change would be demanded. Right now the man on the street is being kept in the dark about money driving medical decisions to the extent it is today including the suppression of medical knowledge if it will interfere with the flow of that money, or some other irrational fear such as troop strength decimation if soldiers were officially offered HBOT by the military to treat their TBI/PTSD.

## Abbreviations

TBI: Traumatic brain injury
ATA: Atmospheres absolute
CTE: Chronic traumatic encephalopathy
PTSD: Post traumatic stress disorder
HBOT: Hyperbaric oxygen therapy
DCS: Decompression sickness
HBO: Hyperbaric oxygen
CP: Cerebral palsy
NFL: National Football League
GMF: Gross motor function
MTBIC: Mild Traumatic Brain Injury Committee
NBIRR: National Brain Injury Rescue and Rehabilitation
DoD: Department of Defense
RCT: Randomized Controlled Trial
FDA: Federal Drug Administration
SSRI: Selective Serotonin Reuptake Inhibitor
MS: Multiple sclerosis

## Footnotes

1 http://clinicaltrials.gov/ct2/show/NCT01105962

2 RAND Report: “Invisible Wounds of War: Psychological and Cognitive Injuries, Consequences, and Services to Assist Recovery.” Tanielian, Terri; Jaycox, Lisa, April 2008, page xxii-xxiii: Two year costs within the first two years the service member returns home; PTSD $5,904 to $10,298 depending on whether we count the lives lost to suicide; Two year costs for major depression, $15,461 - $25,757; co-morbid PTSD and major depression; $12,427 to $16,884; One year costs for traumatic brain injury diagnosis: $25,572 to $30,730 in 2005 for mild cases ($27,259 to $32,759 in 2007 dollars), and $252,251 to $383,221 for moderate or severe cases ($268,902 to $408,519 in 2007 dollars.) These costs, largely treating symptoms, continue to have out year costs and out year consequences in terms of disability payments, inability to work, etc. Given that the HBOT ONE TIME cost for service members who need all 80 treatments averages $16,000 at Medicare Reimbursement rates for a 1-h treatment. HBOT alone, and even HBOT in conjunction with other treatments, is very cost effective. If provided acutely within hours of injury, the treatment is even more effective and massively more cost effective.
